# Gastrosquisis simple complicada con múltiples perforaciones, abdomen congelado y pérdida de dominio abdominal

**DOI:** 10.23938/ASSN.1098

**Published:** 2025-02-13

**Authors:** Diana Cayetano-Cabrera, Cristian Zalles-Vidal, Alejandro Peñarrieta-Daher, Julio César Moreno-Alfonso, Katherine Bautista-Jiménez, Lourdes Melendez-Roque

**Affiliations:** 1 Hospital Infantil de México Federico Gómez Cirugía Pediátrica Ciudad de México México; 2 Hospital Infantil de México Federico Gómez Cirugía Neonatal Ciudad de México México; 3 Servicio Navarro de Salud-Osasunbidea Hospital Universitario de Navarra Cirugía Pediátrica Pamplona España; 4. Universidad Pública de Navarra (UPNA) Programa de Doctorado en Ciencias de la Salud Pamplona España; 5 Hospital Nacional de Niños Benjamin Bloom Cirugía Neonatal San Salvador El Salvador

**Keywords:** Gastrosquisis, Perforación Intestinal, Fístula, Yeyunostomía, Recién Nacido, Gastroschisis, Intestinal Perforation, Fistula, Jejunostomy, Infants, Newborn

## Abstract

La gastrosquisis es una malformación congénita caracterizada por una hernia visceral que representa una de las principales causas de síndrome de intestino corto de las series pediátricas. Puede ser secundaria a intestino corto congénito, pero también derivarse de complicaciones asociadas al manejo del defecto de pared abdominal.

Presentamos el caso de un recién nacido a término con gastrosquisis simple que presentó múltiples complicaciones gastrointestinales adquiridas durante el manejo inicial. Ingresó en nuestra institución con abdomen abierto, congelado, con fístulas entero-atmosféricas y pérdida de dominio abdominal. Durante tres meses se emplearon distintas técnicas combinadas (suturas primarias intestinales, yeyunostomías con realimentación del estoma distal, toxina botulínica, construcción de silo de polipropileno) en respuesta a la aparición de complicaciones hasta la reconstrucción abdominal total. Tras una evolución favorable, el paciente fue dado de alta a los cinco meses de vida, con tolerancia oral y ganancia pondoestatural adecuada.

## Abstract (en)

Gastroschisis is a congenital malformation characterized by a visceral hernia and is one of the leading causes of short bowel syndrome in pediatric patients. This condition can result from congenital short bowel, but may also arise due to complications associated with the management of the abdominal wall defect.

We present the case of a full-term newborn with simple gastroschisis who developed multiple gastrointestinal complications during initial management. On admission in our institution, the patient exhibited an open, frozen abdomen, enteroatmospheric fistulas, and loss of abdominal domain. Over the course of three months, various combined techniques were used, including primary intestinal sutures, jejunostomies with distal stoma feedback, botulinum toxin injections, and the construction of a polypropylene silo, with adjustments made based on the evolving complications. Ultimately, complete abdominal reconstruction was achieved. The patient´s recovery was favorable, and he was discharged at five months of age with oral tolerance and appropriate weight and height gain.

## INTRODUCCIÓN

El Centro Internacional de Información para la Vigilancia e Investigación de Defectos Congénitos define la gastrosquisis como una malformación congénita caracterizada por una hernia visceral, generalmente a través de un defecto de la pared abdominal del lado derecho hasta un cordón umbilical intacto y no cubierto por una membrana^1^. Según el Centro para el Control y Prevención de Enfermedades su incidencia es de 1 cada 1.953 nacimientos^2^. Se clasifica en simple y compleja; esta última se caracteriza por la presencia de atresia intestinal, perforación, necrosis y/o vólvulos, lo que predispone a un pronóstico menos favorable^3^.

La gastrosquisis es una de las principales causas de síndrome de intestino corto de las series pediátricas. Puede ser secundaria a intestino corto congénito pero también derivarse de complicaciones asociadas al manejo del defecto de pared abdominal. Bergholz y col evaluaron en un metanálisis el impacto de los casos complejos sobre la morbilidad y mortalidad a corto plazo, identificando que la mortalidad por gastrosquisis compleja es 7,6 veces mayor que por gastrosquisis simple^3^.

Este caso clínico describe nuestra experiencia en el manejo de un paciente con gastrosquisis simple con múltiples complicaciones adquiridas durante su manejo inicial, que fue derivado a nuestra institución con abdomen catastrófico, séptico y desnutrido, con especial énfasis en la discusión de las diversas estrategias quirúrgicas utilizadas para su resolución.

## CASO CLÍNICO

Este caso clínico se ha desarrollado siguiendo los lineamientos de *The CARE Guidelines: Consensus-based Clinical Case Reporting Guideline Development*^4^.

**Figura 1:**
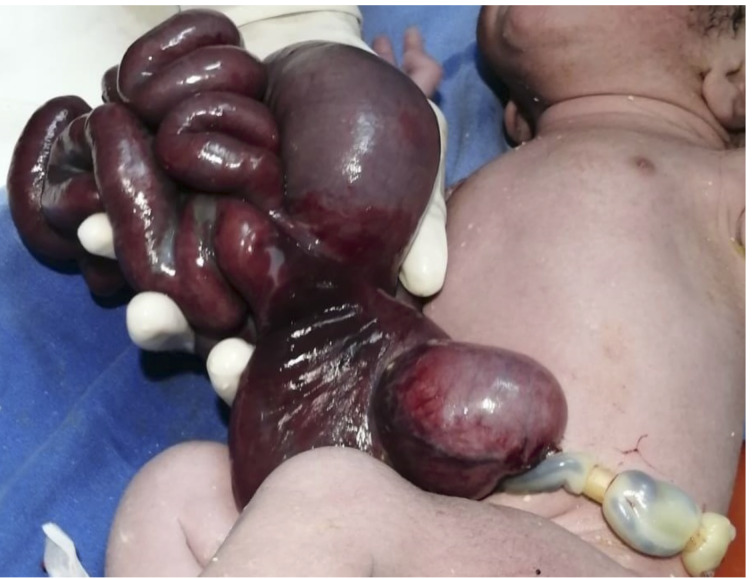
Defecto de pared abdominal a la derecha de cordón umbilical.

Se presenta el caso de un varón de 38 semanas de gestación, nacido por cesárea con Apgar 8/10, 2.950 g de peso y 50 cm de talla, con diagnóstico prenatal de gastrosquisis. Durante la exploración se observó la exposición de asas de intestino grueso y delgado con aspecto violáceo y dilatado, y una desproporción víscero-abdominal evidente (Figura 1). Se calculó una puntuación pronóstica (*Gastrosquisis Prognostic Score)* de 1.

En el hospital de nacimiento se realizó ampliación del defecto el primer día de vida y colocación de silo quirúrgico con bolsa plástica de suero fisiológico suturada a la aponeurosis. A los 11 días de vida, y sin haberse podido reducir el intestino, se observó meconio en el silo, por lo que se indicó la reintervención, durante la cual se observó una perforación intestinal a 70 cm de la válvula ileocecal, realizando resección y anastomosis término-terminal. A los 14 días de vida precisó nueva exploración quirúrgica debido a dehiscencia de la anastomosis, observando un defecto intestinal en la anastomosis de 1 cm de diámetro, con asas friables; se realizó nuevamente resección y anastomosis. Posteriormente, a los 22 días de vida, se observó nuevamente contenido meconial en el silo quirúrgico, por lo que se decidió trasladarlo a nuestro centro hospitalario por falta de opciones terapéuticas en su hospital de referencia.

**Figura 2:**
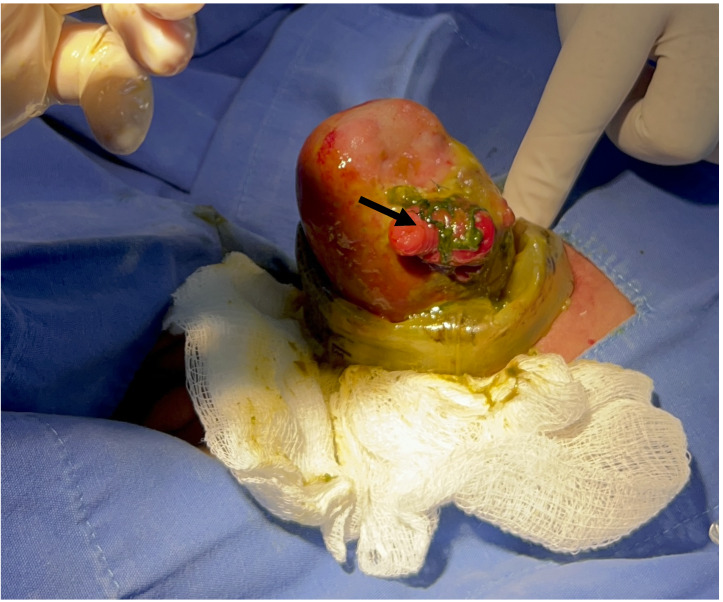
Abdomen congelado con fístulas entero-atmosféricas (flecha negra), a través de las cuales sale meconio.

El paciente ingresó a nuestra institución a los 28 días de vida, con desnutrición severa, con pérdida de 470 gramos respecto al nacimiento y choque séptico con fallo orgánico múltiple, por lo que requirió ventilación mecánica invasiva y soporte vasopresor. A la exploración física presentaba silo desprendido de la pared abdominal, hígado incluido en el defecto, abdomen congelado, peritonitis grave y cinco fístulas entero-atmosféricas (Figura 2). Debido a la gravedad del paciente, al ingreso se priorizó su estabilización respiratoria y hemodinámica, se inició antibioterapia de amplio espectro y nutrición parenteral total.

A los 29 días de vida, se realizó lavado de las asas intestinales expuestas, fistuloclisis con sonda Foley y adhesiolisis leve circunferencial para colocar un silo preformado (Alexis XS®; *Applied Medical*, Rancho Santa Margarita, CA, EEUU), logrando la estabilización del paciente. A los 36 días de vida se aplicaron, bajo control ecográfico, dosis de 20 UI de toxina botulínica (Botox® *AbbVie Spain, S.L.U. Madrid,* España) en cinco puntos diferentes de los músculos oblicuos de cada pared lateral del abdomen, como parte de la preparación para el cierre de la hernia ventral compleja.

A los 38 días de vida se indicó la primera intervención quirúrgica en nuestra institución, buscando una derivación alta para evitar la contaminación abdominal, realizándose adhesiolisis de yeyuno proximal, yeyunostomía de dos bocas a 20 cm del duodeno, resección de dos fístulas entero-atmosféricas y cierre de una tercera en dos planos. Debido a imposibilidad de cerrar la pared abdominal se recolocó un retractor quirúrgico tipo Alexis^®^.

**Figura 3:**
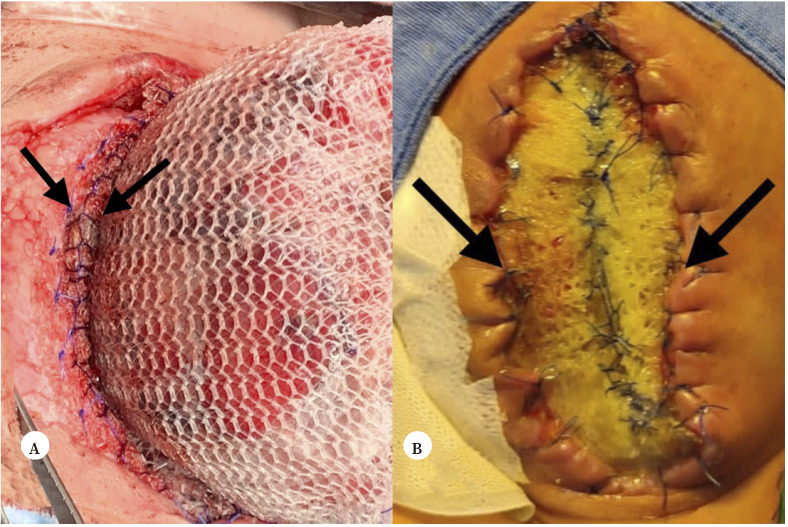
A. Confección de silo con malla de polipropileno (flechas negras) con dos líneas de sutura a cada lado con piel suturada a la primera línea. B. Proceso de reducción del defecto de pared abdominal.

A los 43 días de vida se identificó una lesión en el extremo distal de la yeyunostomía, secundario al anillo del dispositivo Alexis^®^, siendo necesario reintervenir. Se aprovechó este acto quirúrgico para resecar las fístulas restantes y realizar una anastomosis término-terminal. Tras descartar la presencia de atresia intestinal se realizó un procedimiento de Ladd, remodelación del estoma distal de la yeyunostomía e intubación mediante sonda Pezzer^®^ de 10 Fr. Para el cierre de pared abdominal se confeccionó un silo quirúrgico con malla de polipropileno, que fue suturado circunferencialmente a la fascia con dos líneas de sutura para asegurar su sujeción. Previamente a la confección del silo de polipropileno, las asas intestinales fueron protegidas con una bolsa plástica (Figura 3A). A los 44 días de vida se iniciaron las reducciones seriadas del silo mediante surjete continuo anclado, logrando la reducción del contenido abdominal a los 52 días de vida con posterior retirada de la malla y cierre de la pared abdominal sin material protésico (Figura 3B).

La estancia hospitalaria en nuestro centro duró cuatro meses, requiriendo soporte con ventilación mecánica durante 48 días. La recuperación nutricional se llevó a cabo de forma mixta, con nutrición parenteral durante 88 días. La alimentación oral se inició siete días después del cierre de la pared abdominal, requiriendo 23 días para llegar al aporte enteral total. Debido al elevado débito de la yeyunostomía, se decidió brindar alimentación principal por el estoma distal (yeyunostomía intubada) y un mínimo volumen por vía oral. Finalmente, se realizó el cierre electivo de la yeyunostomía a los cuatro meses de vida, dándole de alta a los cinco meses de vida.

Actualmente, con 2 años de vida, el paciente ha evolucionado favorablemente, la pared abdominal se encuentra íntegra, con suficiencia intestinal, ganancia pondo-estatura adecuada e hitos del desarrollo acordes a su edad cronológica.

## DISCUSIÓN

Aun cuando la supervivencia de los pacientes con gastrosquisis en países de ingresos altos es muy buena (97,8% según Fullerton y col^5^), podemos enfrentarnos a casos muy complejos, ya sea por complicaciones congénitas o adquiridas, en los que la mortalidad es incluso 7,6 veces mayor que en aquellos con gastrosquisis simple, de acuerdo con Bergholz y col^4^^,^^6^. Según la *Canadian Pediatric Surgery Network*, los pacientes con diagnóstico prenatal de gastrosquisis, especialmente en los que se sospecha gastrosquisis compleja, deben atenderse en centros que cuenten con experiencia en su manejo^7^; la necesidad de traslado a un centro para su manejo definitivo fue un predictor significativo de peor pronóstico^7^.

Para resolver las múltiples complicaciones adquiridas presentes en este caso, el tratamiento se dividió en cinco fases:

- *La estabilización del paciente* (manejo hemodinámico, nutrición parenteral total y antibiótico);
- *Control de la contaminación abdominal* producida por las fístulas, lo cual se logró inicialmente con el lavado intestinal, fistuloclisis y protección intestinal con dispositivo Alexis® y definitivamente con la yeyunostomía alta. Empleamos como protección intestinal inicial el retractor quirúrgico tipo Alexis®, este es un silo preformado, utilizado en nuestro protocolo de manejo gastrosquisis, el cual empleamos en casos de gastrosquisis simple pero también complejos, con buenos resultados^8^. En este caso el anillo interno del separador lesionó la yeyunostomía distal, lo cual se hubiera podido prevenir si se hubiese realizado la derivación en una posición más lateral;
- *Cobertura de las asas intestinales para reducirlas a la cavidad abdominal*, para lo cual se empleó un silo de Prolene suturado a la aponeurosis. Esta técnica fue descrita por un grupo de cirujanos en el *Great Ormond Street Hospital for Children*, inicialmente para el manejo de los defectos abdominales grandes en gemelos siameses y de onfaloceles gigantes; este silo permite la tracción de los bordes con poco riesgo de desprendimiento del silo^9^^,^^10^. Se han descrito casos en los cuales se utilizó la técnica de construcción de silo a partir de una malla de polipropileno. Bhatnagar y col describieron una técnica sencilla de construcción de un silo utilizando una malla de polipropileno cubierta por ambos lados con una película adhesiva transparente estéril para el tratamiento de 25 casos de gastrosquisis y 13 casos de onfalocele, con resultados aceptables^11^. En un paciente con onfalocele con seguimiento a 5 años, el silo proporcionó una red para reducir el defecto con una adhesión mínima, y fue estable en presencia de inflamación, logrando una evolución favorable y sin complicaciones^12^;
- *Inyección de toxina botulínica en la musculatura de la pared abdominal* como adyuvante a este manejo. Existen múltiples descripciones de su uso en el cierre de hernias gigantes ventrales en adultos, cierres de onfalocele y gastrosquisis^13^. Benchaya y col se plantearon que el uso de toxina botulínica a dosis bajas podría facilitar el tratamiento quirúrgico de hernias incisionales ventrales complejas en niños, ajustando la dosis y los puntos de referencia anatómicos según el tipo de hernia, la edad y el peso del paciente^14^^-^^16^;
- En casos como este donde los pacientes son sometidos a múltiples procedimientos quirúrgicos intestinales, el apoyo de la nutrición parenteral total es esencial, sin embargo, para lograr una adecuada ganancia ponderal y disminuir los efectos secundarios del uso prolongado de esta, utilizamos la realimentación del estoma distal o *puenteo intestinal* (alimentación por yeyunostomía intubada), favoreciendo la resolución de la colestasis, el trofismo del intestino distal y una adecuada recuperación nutricional^17^.

En conclusión, los pacientes con gastrosquisis, en especial las complejas o complicadas, requieren un abordaje multidisciplinar. El equipo médico- quirúrgico al cargo debe contar con diversas estrategias médicas y múltiples herramientas quirúrgicas, como los silos preformados, silos de polipropileno, toxina botulínica y manejo de abdomen catastrófico, para lograr el mejor desenlace posible.

## Data Availability

Se encuentran disponibles bajo petición al autor de correspondencia.
